# Mast Cells and the Pancreas in Human Type 1 and Type 2 Diabetes

**DOI:** 10.3390/cells10081875

**Published:** 2021-07-23

**Authors:** Matilde Masini, Mara Suleiman, Michela Novelli, Lorella Marselli, Piero Marchetti, Vincenzo De Tata

**Affiliations:** 1 Department of Translational Research and New Technologies in Medicine and Surgery, University of Pisa, Via Roma, 55-Scuola Medica, 56126 Pisa, Italy; matilde.masini@unipi.it (M.M.); michela.novelli@med.unipi.it (M.N.); 2 Department of Clinical and Experimental Medicine, Pancreatic Islet Laboratory, University of Pisa, 56124 Pisa, Italy; mara.suleiman@for.unipi.it (M.S.); lorella.marselli@med.unipi.it (L.M.); piero.marchetti@med.unipi.it (P.M.); 3 Centro Interdipartimentale di Microscopia Elettronica (C.I.M.E.), University of Pisa, 56126 Pisa, Italy

**Keywords:** mast cells, type 1 diabetes, type 2 diabetes, pancreatic beta cells, insulin secretion

## Abstract

Mast cells are highly differentiated, widely distributed cells of the innate immune system, that are currently considered as key regulators of both innate and adaptive immunity. Mast cells play a key role in health and survival mechanisms, especially as sentinel cells that can stimulate protective immune responses. On the other hand, it has been shown that mast cells are involved in the pathogenesis of several diseases, and recently a possible pathogenetic role of mast cells in diabetes has been proposed. In this review we summarize the evidence on the increased presence of mast cells in the pancreas of subjects with type 1 diabetes, which is due to the autoimmune destruction of insulin secreting beta cells, and discuss the differences with type 2 diabetes, the other major form of diabetes. In addition, we describe some of the pathophysiological mechanisms through which mast cells might exert their actions, which could be targeted to potentially protect the beta cells in autoimmune diabetes.

## 1. Introduction

**Figure 1:**
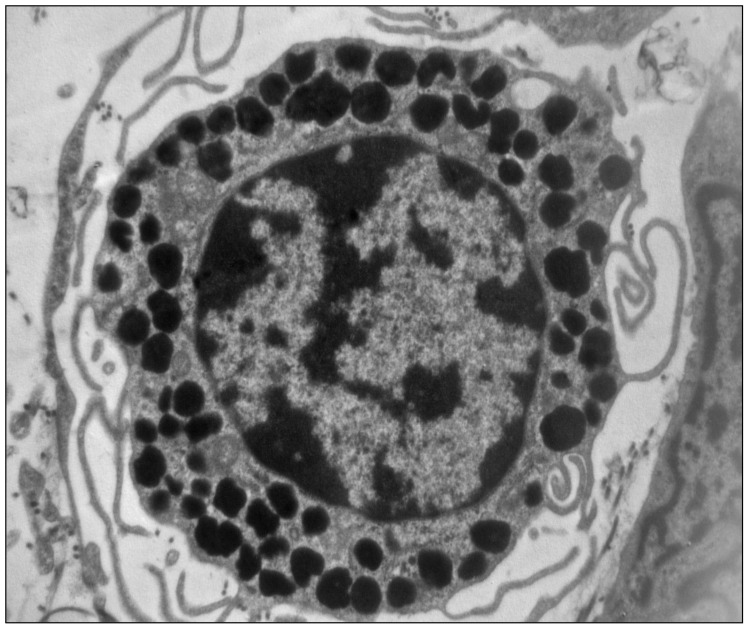
Electron micrography of a mast cell showing the characteristic cytoplasmic granules containing histamine (×10,000) (unpublished image, courtesy of M. Masini).

Mast cells (Figure 1) were first described in 1878 by Paul Ehrlich who observed some granular cells in slides obtained from connective tissue and stained with specific aniline dyes [1]. The word “mast”, derived from the original Greek μαστοσ = breast, was adopted by Ehrlich in relation to the supposed nourishing functions of these cells [1]. Mast cells are among the first immune cells to appear during evolution. Cells showing the characteristic metachromatic staining appeared in urochordates and several fish species more than 500 million years ago, well before the development of adaptive immunity [2]. The remarkable conservation of mast cells throughout evolution is consistent with their importance in innate immunity. These cells are associated with both health maintenance and disease development, and recently a possible role in diabetes has been suggested. In this review we will describe the main morphological, functional, and molecular features of mast cells, with details on their association with pancreas and islet infiltration in type 1 and type 2 diabetes.

## 2. The Mast Cells

Mast cells are terminally differentiated, highly distributed, granular cells of the immune system originating from CD34^+^/CD117^+^ haemopoietic stem cells in the bone marrow [3]. Their progenitors migrate from the bone marrow to the blood first, and then to other tissues, where they further differentiate to mature mast cells under the control of several tissue-specific factors, such as extracellular matrix proteins, adhesion molecules, cytokines, and chemokines [4,5,6]. Recently, Gentek et al. [7] reported that mast cells can have dual developmental origins. In fact, by lineage tracing experiments they showed that mast cells are yolk-sac-derived in the embryo, but are subsequently replaced in the adult by definitive mast cells, which maintained themselves independently from the bone marrow. Mature mast cells are distributed in virtually all tissues, including the brain, but their numbers are particularly elevated at the interfaces with the outside environment, as in the skin and mucosal tissues, where they reside in proximity to nerves, blood, and lymphatic vessels [8]. This wide tissue distribution and the particular localization are functional to the role played by these cells in the innate immunity system. In fact, mast cells are generally considered as sentinel cells, able to sense danger signals, and therefore play a crucial role in the first-line defense against potential pathogens [8].

In both rodents and humans, mast cells appear to be heterogeneous in nature [9,10,11]. Distinct subtypes can be identified on the basis of their structural and functional properties, with particular reference to the neutral proteases expressed within intracellular granules (Table 1). Murine mast cells are classified into two subgroups: connective tissue mast cells (CTMC), which can be isolated from skin, peritoneal cavity and intestinal submucosa, and mucosal mast cells (MMC), which can be found predominantly in the mucosal layers [10]. Two main types of mast cells are generally recognized in humans, according to their protease content: MC_TC_ containing both tryptase and chymase, and MC_T_ expressing only tryptase [11].

Mast cells express an array of activating and inhibitory receptors (mast cells have been claimed to express more receptors than any other cell type) on their surface, which enables these cells to recognize and respond to a wide spectrum of both exogenous pathogens and endogenous molecules derived from damaged or inflamed tissues [12,13]. Mast cells can be activated by either receptor-dependent or independent mechanisms [14]. The best-known activation pathway involves IgE/FcεRI signaling [15]. When IgE bound to high-affinity IgE receptors (FcεRI) encounter a multivalent antigen (or allergen), the receptors on the surface of mast cells are cross-linked or aggregated and the FcεRI signaling is activated. Alternatively, mast cells can also be activated via microbial pattern recognition receptors, such as Toll- and NOD-like receptors (TLRs and NLRs) [16]. More recently, a new Mas-related G protein-coupled receptor X2 (MRGPRX2) has been described, which can recognize cationic neuropeptides, antimicrobial peptides, and insect venom peptides [17]. Furthermore, mast cells can recognize complement, cytokines, and several other stimuli [18].

When activated, mast cells release a large variety of mediators which have been classified on the basis of the kinetics of their release [19,20]. The first group includes preformed granule-stored mediators such as histamine, serotonin, tumor necrosis factor (TNF)-α, proteases such as tryptases and chymases, and proteoglycans. The components of the second group of mediators are released less fast, and include *de novo* synthesized lipid metabolites of arachidonic acid, such as prostaglandins and leukotriens. The third group includes various cytokines and chemokines which are synthesized in response to stimulation through unregulated gene expression.

In view of the large amount of secreted mediators (no other cell is thought to make more mediators), performing a variety of different biological functions, it is not surprising that mast cells are currently considered not simply as effector immune cells, but rather as key regulators of both innate and adaptive immunity [21,22]. It has also been proposed that mast cells, through their ability to release growth factors and cell-specific tryptases and chymases, are involved in tissue remodeling and angiogenesis [22,23]. Mast cells can also play a role in other physiological functions, including organ development [24], wound healing [25], and heart function [26].

Thus, mast cells can be considered key players in health and survival mechanisms, especially as sentinel cells that sense pathogens and stimulate protective immune responses. Indeed, there are no humans without them. On the other hand, mast cells are involved in the pathogenesis of many diseases [27]. In fact, they are primarily known as effector cells in type I allergic reactions and diseases, such as allergic rhinoconjunctivitis, hives, and anaphylaxis [28]. In the development of IgE-dependent type I allergy, the first step is sensitization, during which allergens activate Th2 lymphocytes secreting IL-4, which is essential for the isotype switching from IgM to IgE. IgE are released by plasma cells in the bloodstream and bind to FcεRI receptors in both mast cells and basophils. The subsequent binding of the allergen to IgE already linked to FcεRI receptors on the membrane of mast cells triggers their degranulation and the release of pro-inflammatory mediators responsible for the clinical manifestations of allergy [19].

However, phylogenetic studies showing that mast cells can be found even in animals lacking immunoglobulins, together with the variety of mediators released upon mast cell activation, suggest that these cells could be involved in the pathogenesis of several diseases besides those requiring IgE [28]. In particular, in the last few years, several pieces of evidence have been obtained indicating that mast cells could participate in the pathogenesis of human autoimmune diseases [27,29]. Elevated levels of mast cells have been observed in the inflamed synovium of patients with rheumatoid arthritis, a systemic autoimmune disease mainly affecting synovial joints [30]. At this level, an increased release of mast-cell-derived mediators could contribute to initiate and/or amplify the inflammatory response [31,32]. Moreover, some mast-cell-derived mediators can induce osteoclast differentiation and activation associated with bone destruction [33,34]. In addition, several findings indicate a possible involvement of mast cells in multiple sclerosis, an autoimmune disease affecting the central nervous system (CNS) [35,36]. As a matter of fact, mast cells have been observed in the plaques of multiple sclerosis patients and their amount and distribution correlate with the severity of the disease [37]. Histamine released by mast cells could also facilitate the penetration of autoreactive T cells in the CNS by altering vascular permeability and TNF-α can recruit neutrophils and other inflammatory cells [38]. Moreover, mast cell proteases have been shown to accumulate in the cerebrospinal fluid of multiple sclerosis patients [39] where they can exert a myelinolytic activity [38].

However, in other circumstances, mast cells can contribute to the restoration of homeostasis. In mammals, a positive role of mast cells in inflammation has been identified by using mast-cell-deficient mice as experimental models [27,40]. Other studies have shown that mast cells can help to dampen inflammation induced by toxins, ultraviolet B irradiation, or bacterial infections [41,42,43], possibly due to the secretion of IL-10 [41,42,44].

Therefore, mast cells are sophisticated cells of the innate immune systems, which are able to carry out a wide spectrum of pleiotropic effects, either deleterious or potentially protective.

## 3. Something on Diabetes Mellitus

Diabetes mellitus is a heterogenous disorder of the metabolism of carbohydrate, fat and protein, due to the interplay of genetic and environmental factors [45]. It is characterized by an absolute or relative shortage of insulin production and secretion from the pancreatic beta cells that, together with variable degrees of insulin resistance, leads to hyperglycemia, the clinical hallmark of this condition [45,46,47,48]. The current and most widely accepted classification of diabetes consists of four major categories (Table 2) [45]:

(1) Type 1 diabetes, in most patients caused by autoimmune destruction of pancreatic beta cells (type 1A) and in some cases of non-autoimmune, unknown origin (type 1B or idiopathic, also associated with permanent insulinopenia);
(2) Type 2 diabetes, caused by variable degrees of beta cell functional mass loss, often in the background of reduced insulin sensitivity;
(3) Specific types of diabetes, due to several different causes;
(4) Gestational diabetes.

In type 1 diabetes, which represents up to 10% of all cases, the key pathophysiological feature is the autoimmune destruction of beta cells, mediated by the interaction of innate and adaptive immunity [45,46,47]. In type 2 diabetes, the most common form of this disease (80–90% of all patients), beta cell mass has been reported to be decreased by approximately 30%, with specific insulin secretion functional impairments possibly playing the key role in most cases [48,49,50,51,52].

It has been estimated that the global prevalence of diabetes in 2019 (age 20–79 years) was >9% (463 million people), which is expected to increase to 10.2% by 2030 and 10.9% by 2045 [53]. In addition, about 50% of people with diabetes do not know that they have the disease [53]. A further burden is represented by impaired glucose tolerance, a condition associated with a high risk for the development of the disease. The global prevalence of impaired glucose tolerance was 7.5% in 2019, and is estimated to reach 8.0% by 2030 and 8.6% by 2045.

Morbidity and mortality in diabetic patients are high [47,48,54]. Cardiovascular diseases account for up to more than half of all diabetes-related deaths, diabetic retinopathy is the leading cause of blindness in the working age population, and diabetic nephropathy (alone or in combination with hypertension) causes >80% of end-stage renal disease globally. Because of all this, in 2019 diabetes caused >4 million deaths and demanded almost 800 billion USD in health expenditure [55].

In summary, diabetes is one of the most prevalent non-communicable diseases, carrying an ominous clinical and economic burden. Although heterogenous in its manifestations and mechanisms, diabetes occurs and progresses when pancreatic beta cells fail to produce sufficient amounts of insulin to regulate blood glucose levels and other metabolic processes. Much attention is being therefore pointed to the pathophysiology of insulin-secreting cells in the pancreas.

## 4. Mast Cells in the Pancreas of Type 1 and Type 2 Diabetes

Initial evidence of the presence of mast cells in the pancreas dates back to the late 1950s [56], but more detailed information in humans only appeared several years later [57,58]. In 1971, Westermark studied 23 subjects aged 60 years or older, of whom 12 were non-diabetic and 11 were affected by “maturity onset” diabetes [57]. The author also assessed the role of islet amyloidosis. In non-diabetic individuals without amyloidosis in the islets, intra-insular mast cells were 19.1 ± 3.0 per mm^2^ (mean ± SEM), whereas in those with mild amyloidosis (<2% of the islet area) the number of mast cells within the islets was 31.6 ± 3.3 per mm^2^. In diabetic individuals there was a significantly increased degree of islet amyloidosis and an approximately 3-fold larger number of intra-islet mast cells (61.5 ± 19.5 per mm^2^), which correlated with the extent of islet amyloidosis. Shortly afterwards, it was shown that the increase of mast cells in the pancreatic islets can occur independently from amyloidosis and appears therefore to be a direct consequence of diabetes [58]. Interestingly, sporadic mast cells have also been observed in the inter-acinar connective tissue [58]. In comparison with the intra-islet ones, mast cells within the non-islet tissue were larger and with more densely packed and bigger granules [58]. Successive studies have confirmed the presence of mast cells in the non-endocrine pancreas and discussed their role in acute and chronic pancreatitis, as well as pancreatic cancer [59,60,61]. In pancreatitis, mast cells, activated by an IgE-dependent mechanism and/or by a stem cell factor (SCF)-c-kit autocrine mechanism, are a relevant component of the inflammatory infiltrate. The localization of mast cells close to degenerating acini and regenerating ducts suggests that these cells play a crucial role in both pancreatic tissue destruction and remodeling [60].

**Figure 2:**
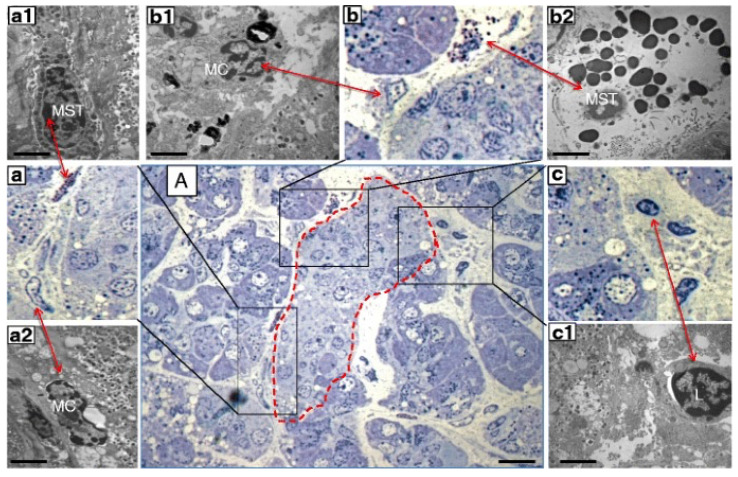
Consecutive semithin and ultrathin sections of a pancreas sample from a donor with type 1 diabetes showing (**A**) a pancreatic islet (dashed line) surrounded by infiltrate containing different inflammatory cells (×1000). The comparison between consecutive semithin and ultrathin sections illustrates how the identification of the different types of inflammatory cells in the semithin section was confirmed by electron microscopy. (**a**–**c**) ×1000 magnification (semithin sections) of the corresponding images in (**A**); (**a1**,**a2**) electron microscopy images of a mast cell (MST) and macrophage (MC) identified in (**a**) (×10,000); (**b1**,**b2**) electron microscopy images of an MC and degranulating MST identified in (**b**) (×10,000); (**c1**) electron microscopy of a lymphocyte (L) identified in (**c**) (×10,000). Scale bars correspond to 10 μm in A, and to 1 μm in (**a1**,**a2**,**b1**,**b2**,**c1**). Reproduced with permission from Ref. [62].

Only recently, however, the role of mast cells in the pancreas of diabetic individuals has been investigated. Martino and colleagues [62] examined pancreatic samples from seven multiorgan donors without diabetes, six with type 1 diabetes and seven with type 2 diabetes, that were prepared for electron and optical microscopy. Mast cells were first studied in semithin sections, stained with toluidine blue and methylene blue. In these samples, mast cells were identified by light microscopy based on their metachromatic staining. Immunohistochemistry evaluations were performed with formalin-fixed, paraffin-embedded samples. A monoclonal mouse antibody against tryptase was used to recognize mast cells. Finally, by electron microscopy mast cells were distinguished based on their typical ultrastructural appearance, comprising monolobed nucleus, surface architecture composed of narrow and elongated folds, the presence of typical cytoplasmic granules and the absence of cytoplasmic glycogen aggregates. The presence of lymphocytes and macrophages was also assessed [62]. The results showed increased infiltration by mast cells in type 1 diabetes (Figure 2). In fact, there were 8.2  ±  2.5 and 18.2  ±  4.3 mast cells/mm^2^ in pancreatic samples from non-diabetic and type 1 diabetic donors, respectively (*p*  <  0.05) However, the number of mast cells in specimens from donors with type 2 diabetes (6.6  ±  1.3 per mm^2^) was similar to that of controls. In further detail, the number of mast cells per islet was more than 3-fold higher in type 1 diabetes (2.0 ± 0.7 vs. 0.6 ± 0.3, *p* < 0.05) than in control samples. Accordingly, the percentage of islets with at least one mast cell was 94% in type 1 diabetic and 27% in non-diabetic individuals (*p* < 0.05). Notably, several mast cells in the samples from type 1 diabetic islets looked partly degranulated (Figure 2), indicating functional activation. In addition, non-islet tissue in the type 1 diabetes pancreata also showed increased mast cell infiltration (18.2 ± 4.3 vs. 8.2 ± 2.5 per mm^2^, *p* < 0.05).

As for the other immune system cells examined, and in comparison with non-diabetic samples, lymphocyte number in pancreatic tissues was significantly greater in type 1 diabetes, and macrophage number was significantly greater in type 2 diabetes [62].

More recently, pancreas samples were obtained from 47 organ donors (16 controls, 16 with type 1 diabetes, 2 with type 2 diabetes, and 13 without diabetes but with autoantibodies (9 single and 4 multiple)) [63]. Electron microscopy was performed to evaluate several ultrastructural features, including immune cell infiltration, and mast cells were identified and subdivided into tryptase-positive and chymase + tryptase-positive cells, based on granule morphology. Tryptase-positive mast cells showed amorphous secretory granules containing cylindrical clusters, whereas chymase + tryptase-positive mast cells had more homogeneous granules [63]. Morphometric analyses showed that the average number of mast cells was the largest, although not statistically significant, in autoantibody-positive and type 1 diabetes donors, in comparison with non-diabetic donors. More precisely, mast cells per 105 μm^2^ tissue were 1.4 ± 0.60 in controls, 2.3 ± 0.49 type 1 diabetic donors (*p* = 0.062 vs. controls), and 2.4 ± 0.57 in autoantibody-positive individuals (*p* = 0.068 versus controls). However, significant differences were observed in the amount of mast cell subtypes. The proportion of tryptase-positive mast cells was >90% of total mast cells in type 1 diabetic donors, and about 50% in autoantibody-positive and control groups. Interestingly, the number of tryptase-positive cells was significantly greater in type 1 diabetic donors (2.2 ± 0.50, *p* = 0.005 vs. controls) and auto-antibody-positive subjects (1.7 ± 0.55, *p* = 0.02 vs. controls) than in non-diabetic control individuals (0.81 ± 0.57). However, and in line with the results of a previous study [62], in the two type 2 diabetes case mast cells were minimally represented [63].

Therefore, the number of mast cells and/or their certain subtypes is increased in the pancreas and the islets of subjects with type 1 diabetes, but not in those of people with type 2 diabetes, suggesting a role of mast cells in autoimmune diabetes.

## 5. Mast Cells in the Pathophysiology of Type 1 Diabetes

Some evidence supports the involvement of mast cells in the pathophysiology of type 1 diabetes. In the BioBreeding (BB) rat, the most extensively studied rat model of type 1 diabetes, it was found that the abundance of mast cells in peripheral lymph nodes is significantly increased and that eotaxin, a chemokine that can recruit mast cells, is expressed in the pancreatic islets, being specifically localized in the beta cells [64,65]. Of interest, treatment with the mast cell degranulation inhibitor disodium cromoglycate significantly delayed the onset of diabetes (from 63 ± 6 to 82 ± 30 days, *p* = 0.045), with 25% of the animals remaining diabetes-free until the end of the study (130 days). Overall, disodium cromoglycate therapy reduced the risk of disease by about one-half, and the animals without diabetes at 130 days had normal islet histology.

A role of mast cells in the pathogenesis of type 1 diabetes has been proposed by some authors in the type 1 diabetes NOD mouse model [66]. In these mice, mast cells are pro-inflammatory and release large amounts of IL-6, which favors differentiation of IL-17-secreting T cells at the site of autoimmunity. At odds with what found in control mice, mast cells from the NOD mice did not undergo tolerogenic differentiation after interaction with FoxP3+ T regulatory cells [66]. However, in another study, in which two strains of mast cell-deficient NOD mice were used (NOD.Cpa3(Cre/+) and NOD.Kit^(W-sh/W-sh)^), it was observed that both incidence and progression of diabetes in this model were independent of mast cells [67]. In the same article, analysis of pancreatic lymph nodes showed that lack of mast cells had no apparent effect on the autoimmune response [68]. At the other end, some evidence suggests that mast cell might even have protective effects on the beta cells. A study used non-diabetic prone mast-cell-deficient mice (W/W(v) or Wsh/Wsh) in which diabetes was induced by multiple low-dose streptozotocin treatment, that causes massive beta cell destruction [67].

Mast-cell-deficient mice developed severe insulitis and rapid progression of hyperglycemia, with 100% of mice becoming diabetic. This was associated with reduced number of T regulatory (Treg) cells in the pancreatic lymph nodes. Furthermore, there was marked reduction of IL-10, TGF-β, and IL-6 in the pancreatic tissue. Of note, mast cell adoptive transfer in this model before streptozotocin-injection-induced resistance to diabetes development, which was associated with increased Treg cells and decreased IL-17-producing T cells [68].

A few sets of data have been generated also with human islets ex vivo. Histamine is one of the most abundant secretory products of mast cells, and its role was tested by direct exposure of isolated human islets to this compound for 72 h [62]. It was observed that the proportion of beta cells with signs of apoptosis increased from the value of 1.0 ± 0.6% in control islets) to 6.1 ± 2.0% in islets incubated with histamine (*p* < 0.01). In addition, the percentage of islet insulin-positive cells showing TUNEL-positive nuclei was significantly greater after incubation with the amine than in control islets (2.8 ± 1.6% vs. 0.6 ± 0.5%). Intriguingly, activation of caspases 9 and 3, key factors in apoptotic processes, was not affected. However, it was found that the expression of apoptosis-inducing factor (AIF, also known as AIFM1) was significantly increased after exposure to histamine [55]. AIF is released from mitochondria in cases of an intrinsic form of caspase-independent apoptosis, to then translocate into the nucleus and mediate DNA damage [69]. The impact of histamine on mitochondria was confirmed by experiments with insulin-producing INS-1E cells, which showed significant inhibition of mitochondrial complex I enzyme activity and depolarization of the mitochondrial membrane potential after 72 h culture with the amine [62].

These discrepant results may be due to the different experimental designs used and/or to the possibility that mast cells might have species specific deleterious or protective actions.

## 6. Conclusions

Mast cells are highly differentiated, widely distributed cells of the innate immune system. The involvement of mast cells in diabetes is corroborated by findings indicating that these cells are associated with inflamed adipose tissue, the development of certain diabetes complications such as diabetic nephropathy, and reduced wound healing in the case of diabetic foot lesions [70,71,72,73]. In the present review we have discussed the role of mast cells in the diabetic pancreas. Their increased presence in the pancreas of human subjects with type 1 diabetes raises the possibility that these cells could be implicated in the pathophysiology of this form of diabetes, which is due to autoimmune destruction of the insulin-secreting beta cells. However, it is not clear if and why mast cells could be dangerous or protective in this regard. More studies are needed to determine whether and how manipulation of these cells might impact on the natural history of type 1 diabetes, which could allow the development of a strategic approach targeted to modulate mast cell function.

## Figures and Tables

**Table 1:** Selected differential characteristics of rodent and human mast cell subsets (modified from Ref. [8]).

**RODENT**		
**Characteristic**	**CTMCs**	**MMC**
Size	Larger (10–20 μm)	Smaller (5–10 μm)
Granule neutral proteases	Chymase, Tryptase, Proteinase 5, Carboxypeptidase A	Chymase
Histamine content	High	Low
**HUMAN**		
**Characteristic**	**MCT**	**MCTC**
Distribution	Predominant subtype in small intestinal mucosa and alveoli	Predominant subtype in skin and small intestinal submucosa
Granule neutral proteases	Tryptase	Tryptase, chymase, carboxypeptidase, cathepsin G

**Table 2:** Current classification of diabetes mellitus (adapted from Ref. [45]).

Category	Essential Features
Type 1 diabetes	Immune-mediated death of pancreatic beta cells (Type 2) Conspicous/absolute insulin deficiency Includes LADA (Latent Autoimmune Diabetes of Adulthood) Includes idiopatic type 1 diabetes (Type 1B) *
Type 2 diabetes	Combination of beta cell dysfunction and death Varying degrees of insulin resistance
Specific types	Heterogenous group Mostly associated with beta cell dysfunction/loss Due to genetic or acquired causes
Gestational diabetes	Onset during pregnancy

* Abandoned by the World Health Organization.

## Data Availability

Not Applicable.
